# Prognostic Impact of Postoperative Systemic Immune-Inflammation Index Changes in Epithelial Ovarian Cancer

**DOI:** 10.3390/cancers17213422

**Published:** 2025-10-24

**Authors:** Young Eun Chung, E Sun Paik, Minji Kim, Na-Hyun Kim, Seongyun Lim, Jun-Hyeong Seo, Chel Hun Choi, Tae-Joong Kim, Jeong-Won Lee, Yoo-Young Lee

**Affiliations:** 1 Gynecologic Cancer Center, Department of Obstetrics and Gynecology, Samsung Medical Center, Sungkyunkwan University School of Medicine, Seoul 06351, Republic of Korea; y9242.chung@samsung.com (Y.E.C.); minjiibee.kim@samsung.com (M.K.); nahyun22.kim@samsung.com (N.-H.K.); sy4577.lim@samsung.com (S.L.); chelhun.choi@samsung.com (C.H.C.); tj28.kim@samsung.com (T.-J.K.); garden.lee@samsung.com (J.-W.L.); 2 Department of Obstetrics and Gynecology, Kangbuk Samsung Hospital, Sungkyunkwan University School of Medicine, Seoul 06351, Republic of Korea; esun.paik@samsung.com

**Keywords:** epithelial ovarian cancer, systemic immune-inflammatory index, prognosis, inflammation

## Abstract

**Simple Summary:**

Ovarian cancer is one of the most serious cancers affecting women, and survival rates remain poor even with surgery and modern treatments. Doctors are increasingly interested in using simple blood tests to understand how the body responds to cancer and surgery. In this study, we looked at a marker called the systemic immune-inflammation index, which reflects the balance between immune cells and inflammation in the blood. We measured this marker before and after surgery in women with ovarian cancer. We found that patients who had a sharp increase in this marker after surgery tended to live for a shorter time without the disease returning and had lower overall survival. These results suggest that monitoring changes in blood-based inflammation after surgery could help doctors identify patients at higher risk and may guide decisions about closer follow-up or additional treatment strategies.

**Abstract:**

Background: Epithelial ovarian cancer is an aggressive malignancy with poor prognosis despite advances in multimodal treatment. The systemic immune-inflammation index (SII) has emerged as a prognostic biomarker in various cancers; however, the impact of surgery-induced inflammatory changes remains unclear. Methods: This study evaluated the prognostic significance of postoperative changes in SII among patients with epithelial ovarian cancer undergoing primary surgery. Data from 374 patients treated at Samsung Medical Center and Kangbuk Samsung Hospital between 2016 and 2021 were retrospectively reviewed. SII was calculated from complete blood counts obtained within one month before surgery and on postoperative day 1. The percentage change in SII was analyzed, and the optimal cutoff was determined using receiver operating characteristic curve analysis. Survival outcomes were assessed using Kaplan–Meier and multivariable Cox regression models. Results: Patients with a postoperative SII increase > 98.4% (Group 2) had significantly poorer overall (HR = 1.86, *p* = 0.009) and progression-free survival (HR = 1.30, *p* = 0.112) compared with those with smaller changes (Group 1). Discussion: High-grade histology, serous subtype, and greater intraoperative blood loss were associated with higher postoperative SII. A marked postoperative increase in SII independently predicted poor survival, suggesting that dynamic inflammatory responses rather than static baseline levels provide additional prognostic information. Conclusions: Perioperative SII monitoring, easily obtainable from routine blood tests, may help identify high-risk patients who could benefit from intensified surveillance or adjuvant treatment. Prospective multicenter studies are warranted to validate these findings and explore whether perioperative modulation of systemic inflammation can improve outcomes.

## 1. Introduction

Epithelial ovarian cancer (EOC) is a leading cause of gynecologic cancer mortality worldwide, with over 300,000 new cases and 200,000 deaths reported globally in 2020 [1]. Despite advances in maximal cytoreductive surgery and the introduction of targeted agents such as PARP inhibitors and bevacizumab, long-term survival remains poor [1,2]. Recent efforts to improve outcomes through combination strategies, including immune checkpoint inhibitors (ICIs), have shown limited benefit, highlighting the need for novel prognostic and therapeutic approaches.

Chronic systemic inflammation promotes cancer progression by altering the tumor microenvironment to support angiogenesis, immune suppression and metastasis [3]. In this context, various blood-based inflammatory markers have been proposed as prognostic tools across malignancies. Among these, the Systemic Immune-Inflammation Index (SII), calculated as platelet count × neutrophil count/lymphocyte count, has recently gained prominence due to its integrative reflection of innate immune activation (via platelets and neutrophils) and adaptive immune suppression (via lymphocytes) [4,5]. Compared to other markers such as Neutrophil-to-Lymphocyte Ratio (NLR) and Platelet-to-Lymphocyte Ratio (PLR), SII offers a more comprehensive evaluation of systemic inflammation [5,6]. Therefore, SII is emerging as a promising inflammation-based biomarker that captures the dynamic interaction between tumor and host immunity. Given its non-invasive, cost-effective and reproducible nature, it holds potential as a practical tool for risk stratification in clinical oncology. Recent meta-analyses have reported that high pre-treatment SII is significantly associated with poorer overall survival (OS) and progression-free survival (PFS) in ovarian cancer patients [4,5]. Thus, SII is also increasingly recognized as a non-invasive, cost-effective, and reproducible biomarker for prognostication and risk stratification in ovarian cancer [6]. Interestingly, several studies in other solid tumors, including colorectal and gastric cancers, have shown that postoperative changes in SII are associated with long-term outcomes, suggesting that surgery-induced inflammatory responses may influence prognosis [7,8,9]. Although cytoreductive surgery in advanced ovarian cancer is expected to induce substantial systemic inflammation, no prior studies have evaluated the prognostic impact of perioperative SII dynamics in this context.

In this study, we aimed to evaluate whether changes in SII before and after primary surgery serve as an independent prognostic factor in patients with epithelial ovarian cancer. We hypothesize that perioperative changes in SII may help identify patients with higher risk of poor prognosis.

## 2. Materials and Methods

### 2.1. Study Population

This retrospective cohort study included patients diagnosed with epithelial ovarian cancer who underwent primary surgery at Samsung Medical Center and Kangbuk Samsung Hospital from 1 July 2016, and 31 December 2021. Eligible patients were those with newly diagnosed advanced-stage epithelial ovarian, fallopian tube, or primary peritoneal cancer who underwent primary surgery, with complete blood count data available both within one month prior to surgery and on postoperative day one.

### 2.2. Data Collection and Variables

Demographic and clinicopathologic data were collected from electronic medical records, including age at diagnosis, FIGO stage, histologic subtype, tumor grade, residual disease status, and biomarkers such as BRCA mutation, mismatch repair (MMR) status and PD-L1 expression. CBC values were obtained at two timepoints: within 30 days prior to surgery and on the first postoperative day.

### 2.3. Systemic Immune-Inflammation Index (SII) Calculation

The SII was calculated using the formula: SII = Platelet count × Neutrophil count/Lymphocyte count. Additional variables included ascites volume and estimated blood loss during the surgery. To evaluate the prognostic significance of perioperative SII changes, the difference in SII (postoperative—preoperative) was calculated.

### 2.4. Cut-Off Determination and Group Classification

To evaluate the discriminative performance of postoperative SII change, receiver operating characteristic (ROC) analysis was performed for death, the area under the curve (AUC) with 95% CI was calculated, and the optimal cutoff point was determined using Youden’s index. Patients were then categorized into two groups: Group 1 (SII difference below the ROC-derived cutoff) and Group 2 (SII difference above the cutoff). This classification was used to explore survival differences and clinical predictors of adverse SII dynamics.

### 2.5. Statistical Analysis

Baseline characteristics were compared between patients in Group 1 and Group 2. Continuous variables (e.g., age, CA-125) are summarized as median with interquartile range (IQR) and compared using the Wilcoxon rank-sum test; if normally distributed, mean ± SD and Student’s *t*-test are additionally reported. Categorical variables, including Charlson Comorbidity Index (CCI), FIGO stage (stage I vs. stage II or above), histological type (serous vs. non-serous), tumor grade (grade 1 vs. grade 2 or 3), germline or somatic BRCA mutation status, MMR status, and PD-L1 status, were compared using the chi-square or Fisher’s exact test, with standardized mean differences used to assess imbalance. Perioperative and postoperative outcomes were analyzed using the same approach.

Overall survival (OS) and progression-free survival (PFS) were estimated by the Kaplan–Meier method and compared with the log-rank test. Prognostic factors were assessed using univariable and multivariable Cox regression, reporting hazard ratios (HRs) with 95% confidence intervals (CIs). The proportional hazard assumption was verified with Schoenfeld residuals. Independent predictors of Group 2 were identified using multivariable logistic regression, with results expressed as odds ratios (ORs) and 95% CIs, and multicollinearity evaluated by generalized variance inflation factor (GVIF).

All analyses were based on available cases, with *p* < 0.05 considered statistically significant. Statistical analyses were performed using R software (version 4.5.1, R Foundation for Statistical Computing, Vienna, Austria).

This study was approved by the Institutional Review Board of Samsung Medical Center (approval no. 2025-09-037) and the Institutional Review Board of Kangbuk Samsung Hospital (approval no. 2025-08-073). The requirement for informed consent was waived due to the retrospective nature of the study.

## 3. Results

### 3.1. Baseline Characteristics

A total of 374 patients were included in the final analysis. To define prognostic groups based on perioperative SII dynamics, a ROC curve analysis was conducted using difference in SII to predict OS. The optimal cutoff value was determined to be 98.4%, yielding the highest Youden Index (0.180), with a sensitivity of 0.740 and specificity of 0.440 (Appendix A). Based on this threshold, patients were divided into two groups: Group 1 (Difference in SII ≤ 98.4%, indicating lower systemic inflammatory response; *n* = 139) and Group 2 (Difference in SII > 98.4%, indicating higher postoperative systemic inflammation; *n* = 216). Group 2 was defined as patients with a postoperative SII increased of ≥ 98.4%, corresponding to roughly a two-fold elevation compared with the preoperative value.

Table 1 summarizes the baseline characteristics of the study population. The two groups were largely comparable in terms of age, FIGO stage, body mass index (BMI), and comorbidities. However, Group 2 showed significantly higher rates of serous histology (serous: 53.7% in Group 1 vs. 66.5% in Group 2, *p* = 0.018) and high-grade tumors (grade 2 or 3: 81.6% in Group 1 vs. 93.0% in Group 2, *p* = 0.002) compared to Group 1. No significant differences were found in Germline or somatic BRCA mutation, MMR, or PD-L1 status between two groups.

### 3.2. Perioperative and Postoperative Outcomes

Surgical approach, surgical complexity, operation time, ascites volume, and estimated blood loss (EBL) were comparable between the two groups (all *p* > 0.05). However, postoperative ICU admission was significantly more frequent in Group 1 compared with Group 2 (17.7% in Group 1 vs. 9.7% in Group 2, *p* = 0.024). Preoperative SII was significantly higher in Group 1 (1084.3 [732.9–2081.7]) than in Group 2 (684.7 [459.8–1069.8], *p* < 0.001). Conversely, postoperative day 1 (POD#1) SII was markedly elevated in Group 2 (2462.3 [1524.1–4453.4]) compared with Group 1 (1364.5 [947.4–2223.0], *p* < 0.001) (Table 2).

### 3.3. Survival Outcomes

Patients were divided into two groups based on the ROC-derived cutoff value (98.4) for the difference between postoperative and preoperative SII values. Group 1 (blue line) includes patients with an SII difference below the cutoff, whereas Group 2 (red line) includes patients with an SII difference above the cutoff. The survival curve demonstrates significantly decreased OS in Group 2 compared to Group 1. Tick marks represent censored observations. The survival difference between the two groups was statistically significant by the log-rank test (*p* < 0.05).

Patients were classified into two groups based on the ROC-derived cutoff value (98.4) for the difference between postoperative and preoperative SII levels. Group 1 (blue line) represents patients with an SII difference below the cutoff, and Group 2 (red line) represents those with an SII difference above the cutoff. The curve shows that patients in Group 2 had significantly shorter PFS compared to those in Group 1. Tick marks indicate censored cases. The difference in PFS between the two groups was statistically significant according to the log-rank test (*p* < 0.05).

**Figure 1:**
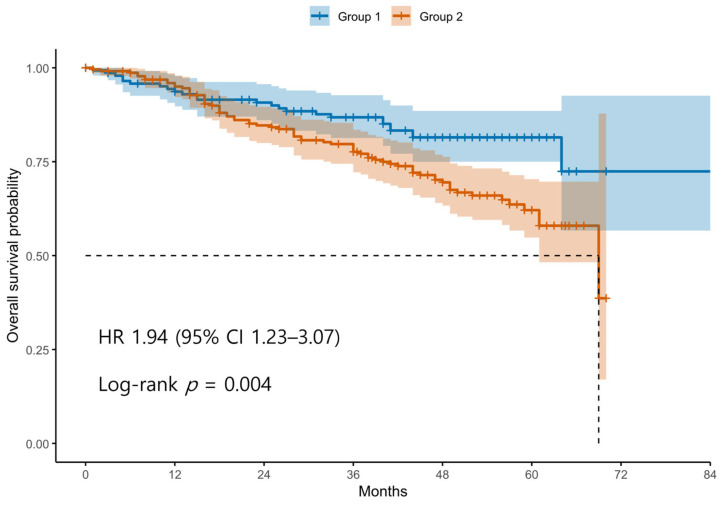
Kaplan–Meier Curves for Overall Survival According to Postoperative SII dynamics. Abbreviations: OS, overall survival; SII, systemic immune-inflammation index; ROC, receiver operating characteristics.

**Figure 2:**
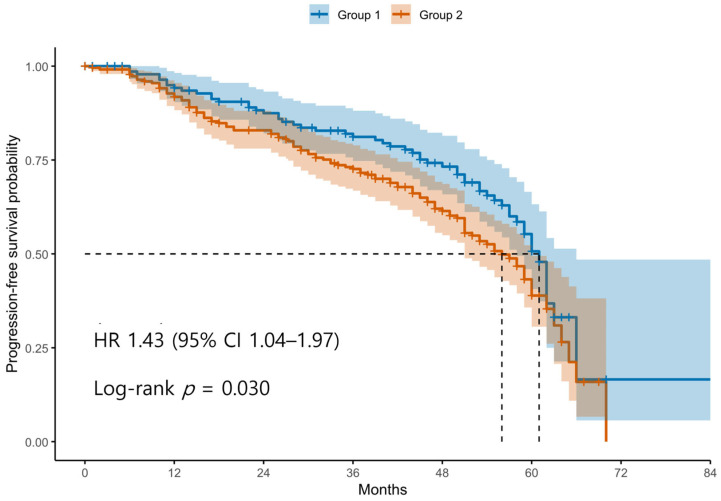
Kaplan–Meier Curves for Progression-Free Survival According to Postoperative SII dynamics. Abbreviations: PFS, progression-free survival; SII, systemic immune-inflammation index; ROC, receiver operating characteristic.

Kaplan–Meier survival analyses revealed significantly poorer OS and PFS in Group 2 compared with Group 1 (Figure 1 and Figure 2). The log-rank test confirmed significant differences between the groups for both OS and PFS (*p* < 0.05 for each). Univariable Cox regression identified several adverse prognostic factors for OS: advanced FIGO stage (HR 6.19, 95% CI 2.87–13.37, *p* < 0.001), higher tumor grade (HR 3.34, 95% CI 1.23–9.12, *p* = 0.016), presence of residual disease (HR 3.71, 95% CI 2.44–5.64, *p* < 0.001), and Group 2 classification (HR 1.94, 95% CI 1.23–3.07, *p* = 0.004). For PFS, advanced FIGO stage (HR 4.76, 95% CI 2.84–7.99, *p* < 0.001), higher tumor grade (HR 5.97, 95% CI 2.21–16.13, *p* < 0.001), residual disease (HR 3.11, 95% CI 2.20–4.40, *p* < 0.001), and Group 2 (HR 1.44, 95% CI 1.04–2.00, *p* = 0.030) were also significant predictors. Notably, non-serous histology was associated with better PFS (HR 0.47, 95% CI 0.33–0.67, *p* < 0.001), although it was not significant for OS (HR 0.74, 95% CI 0.48–1.14, *p* = 0.171).

### 3.4. Prognostic Factor Analysis

In multivariable Cox regression (Table 3), advanced FIGO stage and residual disease remained the strongest independent prognostic factors. For residual disease (Yes vs. No), adjusted HRs were 2.68 (95% CI 1.73–4.17, *p* < 0.001) for OS and 2.08 (95% CI 1.47–2.95, *p* < 0.001) for PFS. Advanced FIGO stage was also independently associated with worse outcomes (OS HR 7.29, 95% CI 3.18–16.70; PFS HR 4.24, 95% CI 2.39–7.51; both *p* < 0.001). Higher tumor grade remained independently associated with poor PFS (HR 4.41, 95% CI 1.60–12.14, *p* = 0.004), although its effect on OS was attenuated (HR 2.33, 95% CI 0.83–6.56, *p* = 0.110). Classification into Group 2 was an independent predictor of worse OS (HR 1.86, 95% CI 1.17–2.97, *p* = 0.009), while its association with PFS did not reach statistical significance (HR 1.30, 95% CI 0.94–1.80, *p* = 0.112). Histological subtype showed divergent patterns: serous histology was independently associated with poorer OS compared with non-serous tumors (HR 2.34, 95% CI 1.46–3.75, *p* < 0.001), whereas for PFS, non-serous histology appeared favorable in univariable analysis (HR 0.47, 95% CI 0.33–0.67, *p* < 0.001), but this association disappeared after adjustment (HR 1.26, 95% CI 0.84–1.90, *p* = 0.260).

### 3.5. Predictors of Elevated Postoperative SII

Multivariate logistic regression was performed to identify independent factors associated with classification into Group 2 (Table 4). Non-serous histology was associated with a lower likelihood of belonging to Group 2 compared with serous tumors (OR 0.40, 95% CI 0.19–0.81, *p* = 0.013). Estimated blood loss < 500 mL was also associated with reduced odds of Group 2 classification (OR 0.28, 95% CI 0.13–0.59, *p* = 0.001). Surgical approach (laparoscopic vs. open) did not reach statistical significance (*p* = 0254). Residual disease status was not retained in this model (*p* = 0.420).

## 4. Discussion

Our analysis demonstrated that an elevated postoperative SII independently predicted poor survival outcomes in patients with epithelial ovarian cancer. Patients in Group 2, who initially presented with lower preoperative SII values, exhibited a disproportionately greater postoperative surge—representing approximately a two-fold increase compared with baseline—which was strongly associated with inferior OS and PFS. These findings highlight that perioperative SII dynamics, rather than static measurements, may provide additional prognostic information. In particular, a marked postoperative surge, rather than stability or reduction, consistently predicted worse survival [10,11]. Our findings therefore add to growing body of evidence suggesting that SII—particularly its perioperative changes—captures not only tumor biology but also the host’s inflammatory response to surgical stress [7,8,9,12].

Interestingly, we observed that patients with serous histology and high-grade tumors were more likely to exhibit pronounced postoperative SII elevations. This suggests that more biologically aggressive subtypes may elicit stronger systemic inflammatory responses, potentially reflecting the interplay between tumor biology and host immunity. Conversely, postoperative ICU admission was more frequent in the group with lower SII increases, underscoring that SII dynamics do not merely reflect perioperative morbidity but rather represent a distinct immunologic signature.

From a clinical standpoint, perioperative SII monitoring offers a widely available tool for risk stratification. Identifying patients with excessive postoperative inflammatory responses may allow for intensified surveillance strategies or consideration of tailored adjuvant approaches. Furthermore, our results raise the intriguing possibility that modulation of perioperative inflammation—through pharmacologic or supportive interventions—could be explored as a strategy to improve outcomes.

Most previous studies have focused on pre-treatment inflammatory markers such as NLR, PLR, or C-reactive protein (CRP), showing consistent associations with poor prognosis in ovarian cancer [11,13,14]. Recent meta-analyses have shown that high preoperative SII is significantly associated with worse survival outcomes [4,5]. In contrast, our study revealed that patients with initially low preoperative SII, but a pronounced postoperative surge (Group 2) also experienced markedly worse OS and PFS. Taken together, these findings suggest that poor prognosis is not confined to patients with elevated baseline SII. A perioperative increase in SII, independent of preoperative levels, represents an additional marker of systemic immune dysregulation and adverse outcomes. While our study did not directly compare SII with other inflammatory markers, prior studies have indicated that SII may offer greater prognostic value than NLR and PLR because it reflects both innate immune activation (via neutrophils and platelets) and adaptive immune suppression (via lymphocytes) [6]. Evidence from gastrointestinal cancers has further shown that persistently elevated perioperative SII correlates with shorter disease-free survival [12,15], and in colorectal cancer, perioperative SII increases predicted higher recurrence and complication rates [9]. Together, these data support the prognostic significance of perioperative SII across malignancies.

The biological plausibility of our findings is underscored by the complex role of postoperative inflammation. While inflammation is necessary for tissue repair, excessive or dysregulated responses may promote a tumor-permissive microenvironment. Surgery induces a cytokine surge (IL-6, IL-8, VEGF), activating neutrophils and platelets while suppressing cytotoxic lymphocytes and facilitating angiogenesis and metastasis [3,16]. A concomitant reduction in lymphocyte count further reflects impaired antitumor immunity, and a high postoperative SII may thus signify tumor-promoting immune dysregulation [17]. Animal and translational studies reinforce this mechanism: murine models demonstrate that surgical stress triggers neutrophil- and platelet-mediated metastatic spread [18], while translational studies show perioperative immune alterations suppress antitumor responses and promote angiogenesis and residual tumor growth [19]. More broadly, systemic inflammation is a recognized determinant of postoperative morbidity and long-term cancer outcomes [20].

An important clinical implication of our findings is the potential to translate perioperative inflammatory dynamics into therapeutic strategies. The perioperative window is increasingly recognized as a period when surgery-related neuroendocrine and inflammatory surges can promote premetastatic processes; conversely, targeted modulation during this interval may mitigate recurrence risk [21,22]. Randomized and translational clinical studies in solid tumors have shown that combined β-adrenergic and COX-2 blockade around the time of surgery favorably alters tumor and immune biomarkers (reduced EMT signatures, favorable immune infiltrates), with promising signals toward lower recurrence in protocol-adherent patients [23] and consistent phase-II data in early-stage breast cancer [24]. Meta-analytic and review data further suggest that perioperative NSAID use may be associated with improved disease-free and overall survival after cancer surgery, including signals in breast and ovarian cancers, although current certainty is limited by heterogeneity and retrospective designs; prospective trials are warranted [25]. Observational data in lung cancer also link perioperative dexamethasone and an NSAID to improved long-term outcomes after resection, supporting the feasibility of anti-inflammatory perioperative strategies [26]. These findings align with a broader body of evidence that perioperative events—including transfusion and excessive inflammatory stress—can adversely affect long-term oncologic outcomes, reinforcing the rationale for risk-adapted, inflammation-sparing pathway alongside pharmacologic modulation [22,27]. Collectively, SII-guided identification of patients with exaggerated postoperative inflammatory surges could help select candidates for such interventions and inform trial design evaluating whether SII-directed perioperative anti-inflammatory therapy improves survival. Beyond these observations, additional epidemiological and preclinical evidence further supports the rationale for perioperative anti-inflammatory therapy in ovarian cancer. Large cohort analyses have demonstrated that regular aspirin or non-aspirin NSAID use is associated with a reduced incidence of epithelial ovarian cancer [28,29]. Laboratory studies have shown that NSAIDs induce apoptosis in ovarian cancer cell lines and suppress NF-κB signaling, thereby enhancing anti-tumor immune responses [30]. These findings, together with meta-analyses showing associations between perioperative NSAID exposure and improved long-term-oncologic outcomes across solid tumors [31], reinforce the concept that systemic inflammatory modulation during the perioperative window may represent a feasible and testable adjunct to standard therapy in ovarian cancer.

The major strength of our study lies in evaluating perioperative changes in SII rather than relying on single pre- or postoperative values. This dynamic assessment may capture the host’s inflammatory and immune response to both surgical stress and tumor removal. In our study, although patients in Group 1 demonstrated higher baseline SII values and a greater need for postoperative ICU admission, these factors did not independently translate into worse overall survival. This observation implies that chronic systemic inflammation or perioperative fragility may reflect short-term clinical risk but may not exert a durable impact on long-term oncologic outcomes. In contrast, a pronounced postoperative rise in SII, as seen in Group 2, appears to represent an acute inflammatory surge that reshapes the tumor–host immune interaction and drives poorer survival. These findings highlight the importance of considering dynamic perioperative changes in systemic inflammation rather than relying solely on static baseline measurements or perioperative complications as prognostic markers. Similar findings have been reported in gastric cancer, where combined analysis of pre-and postoperative LCR (lymphocyte-to-CRP ratio) demonstrated superior prognostic ability compared with either measurement alone [32]. Another distinctive feature is the use of postoperative day 1 (POD#1) SII. Prior studies in endometrial cancer assessed SII on POD#3 [33], and investigations in rectal cancer measured SII at 3–8 weeks after surgery to predict disease-free survival [7]. In our analysis, SII on POD#1 was chosen because it captures the immediate postoperative systemic response before delayed complications occur, providing a consistent time point across patients. Assessing SII as early as POD#1 allowed us to capture the very acute systemic response, before delayed complications such as infection could occur. This early measurement may be practical for rapid postoperative risk stratification, although its prognostic value might partly reflect perioperative factors such as blood loss, fluid shifts, and transfusion. SII on POD#1 may reflect immediate surgical stress; however, its prognostic value persisted after multivariable adjustment, indicating that the observed association is not solely stress-driven

In our analysis, histologic subtype demonstrated divergent associations with survival outcomes. While serous histology emerged as an independent predictor of poor OS, its effect on PFS was less pronounced, and the initially favorable association of non-serous tumors with PFS disappeared after adjustment. This discrepancy may reflect the distinct biological behavior of high-grade serous ovarian cancer, which is characterized by high initial chemosensitivity but frequent recurrence and treatment resistance, ultimately impacting OS more strongly than PFS. Conversely, non-serous subtypes may experience delayed recurrence but lack long-term survival advantage due to limited effective systemic therapies. These findings underscore the need to interpret histology-specific survival patterns in the context of both tumor biology and available treatment strategies.

Several limitations should be acknowledged. First, the retrospective, single-center design may introduce selection bias, and the generalizability of our findings requires validation in large, multicenter prospective cohorts. Second, although the two groups were balanced in most perioperative characteristics, residual confounding by unmeasured variables cannot be excluded. Perioperative transfusion—which has been shown to enhance postoperative systemic inflammation and worsen survival after cancer surgery [27,34], partly through transfusion-related immunomodulation affecting lymphocyte and NK-cell function [35] was not fully incorporated into our model, leaving the possibility of residual confounding. Also, important perioperative confounders such as early postoperative infections, and perioperative use of antibiotics or corticosteroids were not fully adjusted, although previous work suggests that such factors may strongly influence postoperative SII values [36]. Third, SII was measured only on POD#1 as it reflects the acute inflammatory response, but we could not compare with later postoperative time points as reported in other studies [7,33]. Consequently, our study may not fully reflect both the acute inflammatory peak and the subsequent recovery phase. Fourth, we did not directly compare SII with other established inflammatory indices such as NLR, PLR, or CRP; therefore, the relative superiority of SII over other markers cannot be definitively concluded. Lastly, regarding differences in histology and grade between 2 groups, multivariable Cox regression included histologic type and tumor grade to minimize potential confounding associated with these baseline imbalances. Despite these limitations, our study uniquely demonstrates that dynamic perioperative SII changes carry independent prognostic value, underscoring their potential for integration into perioperative risk assessment models.

Future research should focus on developing validated prognostic models that integrate SII dynamics with clinical and molecular features. Prospective, multicenter studies are warranted to confirm the prognostic significance of postoperative SII changes. Additionally, translational research into interventions targeting postoperative inflammation, such as perioperative immunomodulation or anti-inflammatory agents, may provide new avenues to improve long-term outcomes in ovarian cancer. Also, future research should compare SII with established inflammatory markers (e.g., NLR, PLR, CRP) to validate its independent predictive value.

## 5. Conclusions

This study shows that dynamic changes in SII after cytoreductive surgery have significant prognostic value in ovarian cancer. A marked postoperative increase was independently associated with poor OS and PFS, highlighting the relevance of perioperative inflammatory shifts. High tumor grade, serous histology, and greater intraoperative blood loss were significant predictors of heightened postoperative SII, suggesting that both tumor biology and surgical burden contribute to adverse outcomes. Given its simplicity and accessibility from routine blood tests, perioperative SII may serve as a practical biomarker to identify high-risk patients. Prospective validation and interventional trials targeting perioperative inflammation are warranted to confirm these findings and to establish novel therapeutic opportunities.

## Figures and Tables

**Table 1:** Baseline characteristics.

	Total (*n* = 374)	Group 1 (*n* = 147)	Group 2 (*n* = 227)	*p*-Value
Age (years)	54.1 ± 11.6	53.2 ± 12.7	54.6 ± 10.9	0.267
Initial CA-125 (U/mL)	251.5 [42.0, 816.8]	316.1 [42.4, 1184.0]	218.8 [41.7, 706.8]	0.341
Charlson comorbidity index				
Score ≤ 2	304 (81.3%)	120 (81.6%)	184 (81.1%)	0.889
Score ≥ 3	70 (18.7%)	27 (18.4%)	43 (18.9%)	
FIGO stage				
Stage I	105 (28.1%)	42 (28.6%)	63 (27.8%)	0.863
Stage II or above	269 (71.9%)	105 (71.4%)	164 (72.2%)	
Cell type				
Serous	230 (61.5%)	79 (53.7%)	151 (66.5%)	0.013
Non-serous	144 (38.5%)	68 (46.3%)	76 (33.5%)	
Endometrioid	22 (15.3%)	12 (17.7%)	10 (13.2%)	
Clear cell	57 (39.6%)	23 (33.8%)	34 (44.7%)	
Mucinous	43 (29.9%)	22 (32.4%)	21 (27.6%)	
Mixed	10 (6.9%)	6 (8.8%)	4 (5.3%)	
Adenocarcinoma	3 (2.1%)	2 (2.9%)	1 (1.3%)	
Undifferentiated	2 (1.4%)	0 (0.0%)	2 (2.6%)	
Poorly differentiated	3 (2.1%)	0 (0.0%)	3 (4.0%)	
Seromucinous carcinoma	4 (2.7%)	3 (4.4%)	1 (1.3%)	
Grade				
Grade 1	43 (11.5%)	27 (18.4%)	16 (7.0%)	<0.001
Grade 2 or 3	331 (88.5%)	120 (81.6%)	211 (93.0%)	
Germline or somatic BRCA				
Mutated	81 (21.7%)	28 (19.1%)	53 (23.4%)	0.885
Non-mutated	186 (49.7%)	66 (44.9%)	120 (52.9%)	
Not tested	107 (28.6%)	53 (36.0%)	54 (23.7%)	
MMR				
Intact	82 (21.9%)	36 (24.5%)	46 (20.3%)	1.000
Deficient	4 (1.1%)	1 (0.7%)	3 (1.3%)	
Not tested	288 (77.0%)	110 (74.8%)	178 (78.4%)	
PDL1				
Negative	35 (9.4%)	14 (9.5%)	21 (9.3%)	0.596
Positive	48 (12.8%)	22 (15.0%)	26 (11.5%)	
Not tested	291 (77.8%)	111 (75.5%)	180 (79.2%)	

Abbreviations: SII, systemic immune-inflammation index; CA-125, cancer antigen 125; FIGO, International Federation of Gynecology and Obstetrics; BRCA, breast cancer susceptibility gene; MMR, mismatch repair; PD-L1, programmed death-ligand 1. Categorical variables (e.g., surgical approach, SCS, ICU admission) are presented as frequency with percentage *n* (%) and compared using the χ^2^ test or Fisher’s exact test as appropriate. Continuous variables (e.g., ascites volume, estimated blood loss [EBL], pretreatment systemic immune-inflammation index [Pretx_SII], and postoperative day 1 systemic immune-inflammation index [POD1_SII]) are expressed as median with interquartile range (IQR) and compared using the Mann–Whitney U test. In the subgroup of patients with FIGO stage I disease, the distribution of surgical optimality (0/1/2) was additionally compared between the groups.

**Table 2:** Perioperative and postoperative outcomes.

	Group 1 (*n* = 147)	Group 2 (*n* = 227)	*p*-Value
Approach			
Laparoscopic (*n* = 46)	22 (15%)	24 (10.6%)	0.206
Open (*n* = 328)	125 (85%)	203 (89.4%)	
Surgical optimality (Among stage II or above) (*n* = 257)			
No gross residual (*n* = 200)	82 (78.1%)	118 (72%)	0.472
Residual < 1 cm (*n* = 4)	2 (1.9%)	2 (1.2%)	
1 cm ≤ Residual (*n* = 65)	21 (20%)	44 (26.8%)	
Surgical complexity score			
Score ≤ 3 (*n* = 141)	57 (38.8%)	84 (37%)	0.730
Score ≥ 4 (*n* = 233)	90 (61.2%)	143 (63%)	
Operation time (minutes)	230 [175, 281]	228 [169, 302]	0.703
Post-operative ICU care (*n* = 48)	26 (17.7%)	22 (9.7%)	0.024
Ascites (mL)	0 [0, 250]	0 [0, 300]	0.062
Estimated blood loss (mL)	450 [200, 1000]	400 [250, 710]	0.469
Preoperative SII	1084.3 [732.9, 2081.7]	684.7 [459.8, 1069.8]	<0.001
POD#1 SII	1364.5 [947.4, 2223.0]	2462.3 [1524.1, 4453.4]	<0.001

Categorical variables (e.g., surgical approach, SCS, ICU admission) are presented as frequency with percentage *n* (%) and compared using the χ^2^ test or Fisher’s exact test as appropriate. Continuous variables (e.g., ascites volume, estimated blood loss [EBL], pretreatment systemic immune-inflammation index [Pretx_SII], and postoperative day 1 systemic immune-inflammation index [POD1_SII]) are expressed as median with interquartile range (IQR) and compared using the Mann–Whitney U test. In the subgroup of patients with FIGO stage I disease, the distribution of surgical optimality (0/1/2) was additionally compared between the groups.

**Table 3:** Univariable and Multivariable analysis for OS and PFS.

Variables	Overall Survival				Progression Free Survival			
	Univariable HR	*p*-Value	Multivariable HR	*p*-Value	Univariable HR	*p*-Value	Multivariable HR	*p*-Value
FIGO stage (II or above vs. I)	6.19 (2.87–13.37)	<0.001	7.29 (3.18–16.70)	<0.001	4.75 (2.87–7.85)	<0.001	4.24 (2.39–7.51)	<0.001
Histological type (Non-serous vs. Serous)	0.74 (0.48–1.14)	0.171	2.34 (1.46–3.75)	<0.001	0.47 (0.33–0.67)	<0.001	1.26 (0.84–1.90)	0.260
Tumor grade (Grade 2 or 3 vs. Grade 1)	3.34 (1.23–9.12)	0.018	2.33 (0.83–6.56)	0.110	6.10 (2.26–16.46)	<0.001	4.41 (1.60–12.14)	0.004
Residual disease (Yes vs. No)	3.71 (2.44–5.64)	<0.001	2.68 (1.73–4.17)	<0.001	3.13 (2.23–4.39)	<0.001	2.08 (1.47–2.95)	<0.001
Group 2 vs. Group 1	1.94 (1.23–3.07)	0.004	1.86 (1.17–2.97)	0.009	1.43 (1.04–1.97)	0.030	1.30 (0.94–1.80)	0.112

Abbreviations: OS, overall survival; PFS, progression-free survival; HR, hazard ratio; CI, confidence interval; FIGO, International Federation of Gynecology and Obstetrics; SII, systemic immune-inflammation index. Categorical variables (e.g., surgical approach, SCS, ICU admission) are presented as frequency with percentage *n* (%) and compared using the χ^2^ test or Fisher’s exact test as appropriate. Continuous variables (e.g., ascites volume, estimated blood loss [EBL], pretreatment systemic immune-inflammation index [Pretx_SII], and postoperative day 1 systemic immune-inflammation index [POD1_SII]) are expressed as median with interquartile range (IQR) and compared using the Mann–Whitney U test. In the subgroup of patients with FIGO stage I disease, the distribution of surgical optimality (0/1/2) was additionally compared between the groups.

**Table 4:** Multivariate logistic regression analysis of clinicopathologic factors associated with classification into Group 2.

	Odds Ratio (95% CI)	*p*-Value
Histological type (Non-serous vs. Serous)	0.40 (0.19–0.81)	0.013
Tumor grade (Grade 2 or 3 vs. Grade 1)	2.04 (0.63–6.99)	0.238
Residual disease (Yes vs. No)	1.45 (0.59–3.71)	0.420
Surgical approach (Laparoscopic vs. Open)	1.94 (0.61–6.11)	0.254
Estimated blood loss (< 500 mL vs. ≥ 500 mL)	0.28 (0.13–0.59)	0.001

**Figure A1:**
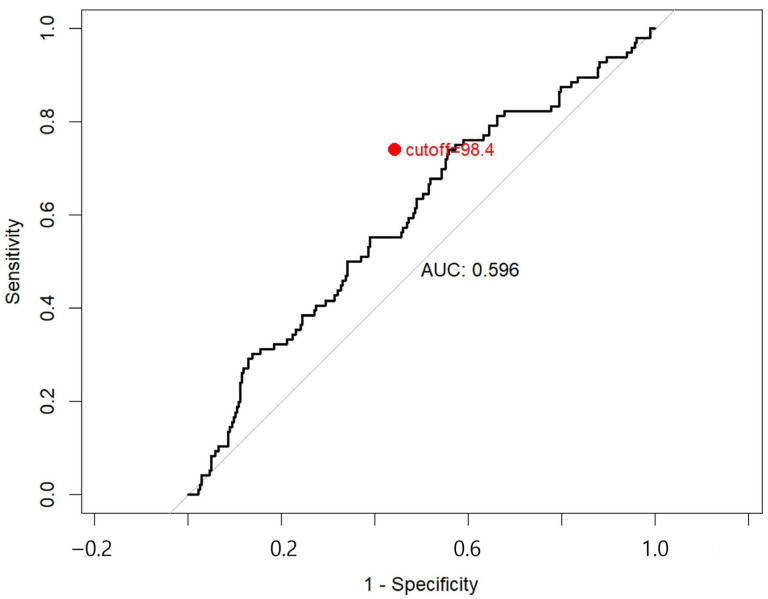
The red dot denotes the optimal cutoff point, determined to be 98.4%, which maximized the Youden index (sensitivity + specificity-1). This threshold was used to stratify patients into Group 1 (ΔSII < 98.4%) and Group 2 (ΔSII ≥ 98.4%) for survival analysis. Abbreviations: ROC, receiver operating characteristic; SII, systemic immune-inflammation index; ΔSII, postoperative change in SII; AUC, area under the curve.

## Data Availability

No new data were created or analyzed in this study. Data supporting the findings of this study are available within the article.

## Abbreviations

The following abbreviations are used in this manuscript:

BRCA	Breast cancer susceptibility gene
CA-125	Cancer antigen 125
CCI	Charlson comorbidity index
CI	Confidence interval
CRP	C-reactive protein
EOC	Epithelial ovarian cancer
FIGO	International Federation of Gynecology and Obstetrics
HR	Hazard ratio
ICI	Immune checkpoint inhibitor
IQR	Interquartile range
MMR	Mismatch repair
NLR	Neutrophil-to-lymphocyte ratio
OS	Overall survival
PD-L1	programmed death-ligand 1
PFS	Progression-free survival
PLR	Platelet-to-lymphocyte ratio
ROC	Receiver operating characteristic
SII	Systemic immune-inflammation index
